# 179 Early Sepsis Identification Using a Host-Response RNA Biomarker in Triaged ED Patients

**DOI:** 10.1017/ash.2026.10725

**Published:** 2026-06-23

**Authors:** Daniel Muleta, Carissa Wallace, Sarah Petersen, Olivia Denzie, Christopher Wilson, Samantha Mathieson

**Affiliations:** 1 Tennessee Department of Health; 2 TENNEESE DEPARTMENT OF HEALTH; 3 TN Department of Health

## Abstract

**Background:** Carbapenem-resistant Acinetobacter baumannii Complex (CRAB) is associated with severe infections and high death rates. The 2024 bacterial priority pathogens list (BPPL) published by World Health Organization indicates CRAB to be a critical priority. This study attempts to describe the 30-day and 90-day mortality following date of specimen collection, and factors associated with higher death rate related to CRAB infections. Method A case was defined as a patient with Acinetobacter baumannii complex isolated from normally sterile sites, urine, lower respiratory tract specimens or wound specimen resistant to meropenem, imipenem or doripenem from the surveillance area. The surveillance area included Davidson and seven other surrounding counties in Tennessee. Epidemiologic and clinical data were collected through the Multi-site Gram-Negative Surveillance Initiative’s (MuGSI) from 2014–2024. Chi-square test was used comparing categorical variables, and logistic regression was used to calculate adjusted odds ratios of variables considered to be associated with mortality. Data analysis was done using SAS 9.4. Result Of 249 cases of CRAB, 151(60.64%) were male; mean age was 60.9 years. There were 167 (67.1%) white, 77 (30.9%) African American and 5 (2.0%) other races. 211(84.74%) cases had isolates from urine whereas 38 (15.26%) were from normally sterile sites. Thirty-seven cases (14.9%) and 60 (24.1%) cases died within 30 days and 90 days of specimen collection respectively. A higher proportion of African American persons died within 30 days compared to White persons (21.3% vs. 12.9%; P Conclusion In this study, older patients had a significantly higher risk of dying and African Americans also appeared to have increased risk of dying within 30 days after specimen collection. The risk of dying within 90 days was much higher for invasive infections. Understanding the risk factors associated with higher mortality is important to improve clinical outcome among patients infected with CRAB.

